# Uncontained spread of Fusarium wilt of banana threatens African food security

**DOI:** 10.1371/journal.ppat.1010769

**Published:** 2022-09-22

**Authors:** Anouk C. van Westerhoven, Harold J. G. Meijer, Michael F. Seidl, Gert H. J. Kema

**Affiliations:** 1 Wageningen University, Laboratory of Phytopathology, Wageningen, the Netherlands; 2 Utrecht University, Department of Biology, Theoretical Biology and Bioinformatics, Utrecht, the Netherlands; 3 Wageningen Research, Department Biointeractions and Plant Health, Wageningen, the Netherlands; Shanghai Center for Plant Stress Biology, CHINA

## Why is banana among the most vulnerable crops?

**Fig 1:**
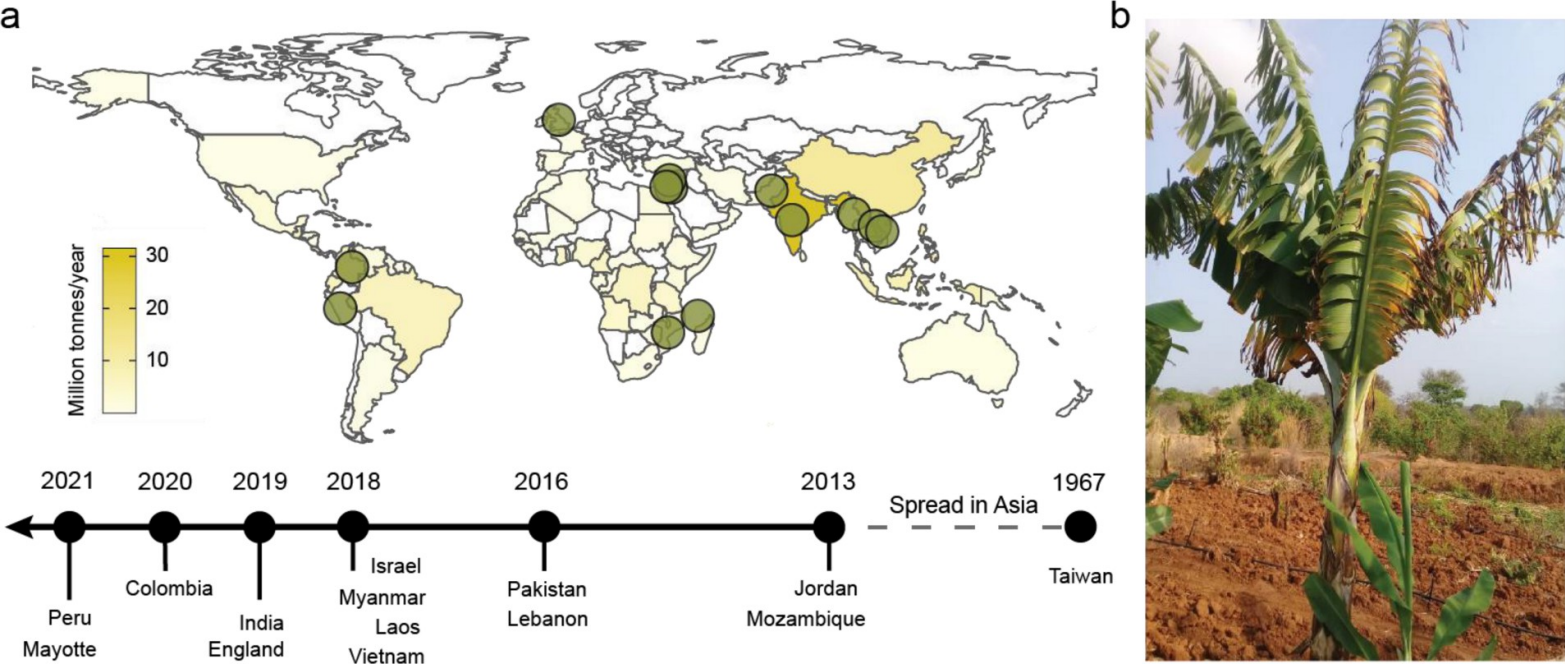
Uncontained spread of Fusarium wilt in banana caused by *Fusarium odoratissimum* TR4. (**A**) Banana is a major food crop in tropical and subtropical regions, especially in sub-Saharan Africa. In most major banana-producing regions, TR4 incursions have been reported (green dots), and TR4 is spreading globally from its Asian center of origin to other banana-growing regions [8,19–27]. The colors of the countries on the global map indicate banana production in million tonnes per year. Map downloaded from Natural Earth Data; https://www.naturalearthdata.com. (**B**) Cavendish banana plant in Mozambique showing external FWB symptoms, caused by TR4 [27].

Banana is the most popular fruit worldwide [1] and a major staple food in tropical and subtropical regions where the majority of bananas is produced (**Fig 1**) [2]. The importance of banana for food security is particularly relevant for East Africa (Burundi, Congo, Rwanda, Tanzania, and Uganda). Here, the East African Highland bananas (EAHBs) are the crucial cash crops and staple food for millions of people with the world’s highest per capita banana consumption of 400 to 600 kg [3]. Throughout this region, banana cultivation is embedded in complex mixed cropping systems by numerous small-scale farmers and households [4]. Most edible bananas are seedless parthenocarpic diploids and triploid hybrids derived from the wild banana species *Musa acuminata* and *Musa balbisiana* [5]. Although the wild, seeded bananas are genetically very diverse [5], the domestication of seedless and hence edible banana varieties resulted in a genetic bottleneck that limits genetic variation [5]. On the local market, different clonal banana varieties are sold, in contrast to the global banana trade that is dominated by clonal Cavendish varieties [6]. These large banana monocultures are extremely vulnerable to numerous diseases [7].

A major concern for banana production is Fusarium wilt, a devastating vascular disease that withers banana plants (**Fig 1**). It is caused by soil-borne fungi belonging to the *Fusarium oxysporum* species complex. Strains that are able to infect banana were known as *F*. *oxysporum* f.sp. *cubense* (*Foc*), despite their well-known diversity [8]. Recently, genotyping analyses confirmed several genetically distinct *Foc* lineages that were consequently recognized as individual *Fusarium* species [9]. For instance, the *Foc* Race 1 strains, which drove a major epidemic of Fusarium wilt of banana (FWB) that almost eradicated the Gros Michel variety dominating the banana trade up to the 1960s in the last century [8], actually comprise a suite of different *Fusarium* species [9]. Eventually, the resistant Cavendish clones saved the industry, and nowadays, 98% of the export market depends on them [6]. However, already in 1967, FWB affected Cavendish in Taiwan [8] (**Fig 1**). The causal *Fusarium* strain, referred to as Tropical Race 4 (TR4), has recently been described as the new species, *Fusarium odoratissimum* [9]. This modified nomenclature of FWB causing *Fusarium* spp. raised some controversy [10], and therefore awaits additional conclusive data. Most experts, however, agree that TR4 is a clonal lineage and genetically so dissimilar from other banana infecting *Fusarium* spp. that it is justifiably recognized as a new species. Next to Cavendish cultivars, TR4 affects a wide range of banana germplasm, including locally important varieties, such as the aforementioned EAHBs [11]. The latter are essential for food security in the African Great Lakes Region where banana is a major staple crop that already suffers from manifold other pests and diseases, such as nematodes, weevils, *Xanthomonas* bacterial wilt, and black leaf streak disease, also known as Black Sigatoka.

## How to respond to a Fusarium wilt incursion?

No commercially available banana variety is resistant to TR4, and consequently, surveillance and disease management are currently the only strategies to control its further dissemination. Traditionally, TR4 incursions were identified based on visual wilting symptoms in Cavendish plants in combination with vegative compatability group (VCG) testing. During this procedure, a nitrate nonutilizing (nit) mutant of a fungal isolate is grown on a Petri dish with known tester strains to assess the ability to form a stable heterokaryon and hence score its compatibility. Such strains are grouped into the same VCG, and TR4 is categorized as VCG 01213 (sometimes also referred to as VCG 01216 [12]). However, this procedure is time-consuming and does not yield reliable results [13]. To curb these disadvantages, a diagnostic PCR was developed and commercialized [14]. Later, other diagnostics became available [15,16], including a fast and easy LAMP test, targeting a different genomic region, which enables rapid identification of TR4 even under field conditions [17]. However, sooner or later, every diagnostic will retrieve false positives, such as by a non-TR4 strain that nevertheless tests positive [18]. Therefore, multiple diagnostics should be used that target different genomic areas for confirmatory reasons. Furthermore, they require continuous monitoring of reliability and consequently updates once false positives are observed, and they should only be evaluated with the biological material for which they were developed. In addition to quick molecular diagnostics, sequencing technologies have made VCG testing redundant as they provide the required resolution to determine the homogeneity and phylogeography of TR4 dissemination. Notably, the genome sequences of various TR4-isolates sampled from independent incursions worldwide reveal very little genetic variation, suggesting its clonal origin ([19]; S1 Dataset). The improved identification and tracing of TR4 are expected to enable rapid implementation and refinement of containment strategies. Nevertheless, FWB caused by TR4 is swiftly spreading across many banana-growing countries worldwide (**Fig 1**).

## Are there options for continued banana production after a Fusarium incursion?

Upon the first reports in Taiwan [8], TR4 disseminated across South East Asia [19]. In 2013, the first incursion outside South East Asia was reported in Jordan [20] (**Fig 1**). Since then, 12 incursions followed in the Middle East [21], the Indian subcontinent [22], Africa [23,24], and most recently in Latin America [25–27] (**Fig 1**).

The arrival of TR4 in Mozambique in 2013 is highly significant due to the importance of bananas as a staple crop in sub-Saharan Africa. Presumably, the TR4 incursion was restricted to 2 commercial plantations in the North of the country [28]. The plantations were placed under quarantine [28] but production was continued, partly with GCTCV218, a less susceptible Cavendish mutant [29]. During surveys in 2015, no suspicious wilting symptoms were detected outside the farms; hence, TR4 was declared to be under control [28]. However, recently, wilting symptoms were observed outside the farm boundaries and subsequent analyses confirmed the dissemination of TR4 to other distant locations (**Fig 1**) [30]. A comparison of 5 fungal strains, isolated from FWB symptomatic banana plants at various locations, to the TR4 II5 reference isolate clearly confirmed TR4 as the causal organism [30]. The analyzed isolates show little genetic diversity [30], suggesting that local transmission occurs through a single clonal lineage. However, the isolates could not be linked to other worldwide reported TR4 incursions since sequencing data typically only include isolates from the first official disease reports that lack sampling depth to address local diversity and dissemination ([30]; S1 Dataset). Consequently, comparative analyses of the genomes from new versus previous incursions enables only provisional associations. The accumulation of unique genetic variation across TR4 strains in Mozambique suggests an extended time of local spread [30]. We, therefore, can neither robustly link the origin of TR4 in Mozambique with incursions in other countries, nor declare the newly discovered TR4 strains as independent novel incursions. We even cannot link them with the 2 initially infested farms because there are no publicly available sequencing data from the strains originally identified at these farms [30]. However, the proximity of the sampling sites to these farms and the applied disease management practices strongly suggest that TR4 was not successfully contained. Moreover, TR4 was recently reported on Mayotte, an island in the Indian Ocean approximately 700 km from the infested farms in Mozambique [24]. Again, the origin of this incursion is unknown due to lacking sequencing data. This underscores the importance of sequencing efforts and rapid data sharing to unveil whether disease management efforts were effective [31].

Taken together, it is very likely that the spread of TR4 in Mozambique was not stopped by cultivating less susceptible Cavendish (GCTCV) somaclones. On the contrary, it is conceivable that this management practice contributed to the further dissemination of TR4. Reduced susceptibility of alternative banana germplasm seems inadequate to manage FWB. Complete resistance is required as shown by the Cavendish varieties that are globally cultivated on Race 1–infested soils for over 70 years without any decline of resistance [8]. Any level of resistance to TR4 that does not meet this standard is insufficient and can contribute to further dissemination of TR4.

## Can FWB caused by TR4 be stopped?

FWB management strategies currently aim to prevent the spread of TR4 by focusing on the use of clean planting material and machinery and the quarantining of infested farms [13,28,32]. However, the ongoing global spread shows that FWB successfully disseminates despite extensive prevention strategies (**Fig 1**). The case study of Mozambique is not the only example of the challenges associated with the containment of TR4 following an initial incursion. The spread of TR4 to Colombia is yet another case. Upon the first report of TR4 in the Guajira department in 2019 [25], Colombia declared a state of emergence entailing sanitary control measures as well as aerial and on-the-ground inspections as part of the containment strategy [32]. Nevertheless, in 2021, TR4 also appeared in the neighboring Magdalena department [27], demonstrating the spread of TR4 irrespective of the implemented containment strategies. A recent incursion in Peru [26] illustrates ongoing local and global spread but is considered to be independent of the presence of TR4 in Colombia [27]. However, this can only be concluded after sufficient sampling in Colombia. These collective data underscore the failure of contemporary management strategies for TR4. The uncontained spread that drives the TR4 pandemic is reminiscent of the previous dissemination of Race 1 strains [7,8] and puts regions at risk that rely on bananas. Notably, the documented spread of TR4 largely involves commercial banana farms. However, the unsuccessful disease management at these large farms raises the concern for small-scale farms that dominate African banana production. Smallholders are frequently disconnected from extension and cannot afford or are less skilled in disease and pest management [4]. Hence, the occurrence of TR4 outside major farms is largely unattended, facilitates its spread, and directly threatens income and food security.

The risks posed by emerging and spreading plant pathogens are increasingly recognized [33]. Like FWB, various plant pathogens affect important food crops. For example, wheat blast, caused by *Magnaporthe oryzae* pathotype *Triticum*, originates from Brazil [34] but destroyed 50% of wheat crops after it emerged in Bangladesh in 2016 and was recently detected in Zambia [35]. Next to their impact on agriculture, invasive fungal diseases on plants and animals can also endanger natural ecosystems [36]. For example, the damage to forests by ash dieback (caused by *Hymenoscyphus fraxineus*) in Europe affects biodiversity and accounts for losses in fixed CO_2_ [37]. Similarly, the fungal pathogen *Batrachochytrium dendrobatidis* causes a significant decline in amphibian populations [36]. Human factors such as increased international travel or environmental and climate changes likely drive pathogens’ emergence, evolution, and dissemination to novel geographic regions or ecological niches [33]. Often new incursions remain unnoticed and once fungal pathogens are endemic, successful disease management is basically unfeasible, as exemplified by the very few examples of successful eradication [38,39]. Such cases often rely on fungicides and thorough eradication of host plants, illustrating the importance of an accurate understanding of the host range of a pathogen [39]. Effective and open science at local and global scales are indispensable to enable a rapid and coordinated response to emerging and invasive fungal diseases [31]. TR4 continues to disseminate (**Fig 1**), irrespective of implemented strategies, and we observe that new incursions often do not lead to effective and transparent responses and data sharing, which are required to improve disease control. The recently reported uncontrolled dissemination of FWB in Mozambique [30] is a serious threat to African food security and global banana production. Now, nearly 10 years after its introduction to Africa, we call for radical eradication strategies of TR4, along with proactive screening for resistance of African banana germplasm and intensified breeding programs for this important staple crop.

## Supporting information

S1 Dataset Overview of the samples used in the study. (XLSX)
